# Randomised controlled trial: nutritional supplements to relieve irritable bowel syndrome symptoms by targeting the gut microbiota

**DOI:** 10.1017/jns.2025.10021

**Published:** 2025-07-11

**Authors:** Maartje van den Belt, Zhuang Liu, Lonneke Janssen Duijghuijsen, Erwin G. Zoetendal, Ben Witteman, Nicole M. de Roos, Paul Vos, Hauke Smidt, Nicole J.W. de Wit

**Affiliations:** 1 Wageningen Food & Biobased Research, Wageningen University & Research, Wageningen, The Netherlands; 2 Laboratory of Microbiology, Wageningen University & Research, Wageningen, The Netherlands; 3 Division of Human Nutrition and Health, Wageningen University & Research, Wageningen, The Netherlands; 4 Gastroenterology and Hepatology Department, Hospital Gelderse Vallei, Ede, The Netherlands

**Keywords:** Bifidobacterium, Functional foods, Irritable bowel syndrome, severity, Microbiota, BMI, Body Mass Index, EMA, Ecological Momentary Assessment, FDR, False Discovery Rate, FFQ, Food Frequency Questionnaire, HADS, Hospital Anxiety and Depression Score, IBS, Irritable bowel Syndrome, IBS-C, IBS with predominant constipation, IBS-D, IBS with predominant diarrhoea, IBS-M, IBS with a mix of constipation and diarrhoea, IBS-U, IBS with no specific stool pattern, IBS-SSS, Irritable Bowel Syndrome Severity Scoring System, ITT, Intention To Treat, PCR, Polymerase Chain Reaction, PP, Per protocol, SCFA, short-chain fatty acids, QoL, quality of life

## Abstract

In individuals with irritable bowel syndrome (IBS), eliminating dietary triggers can alleviate symptoms but may lead to nutrient deficiencies and overall health decline. Although various nutritional supplements show promising results in relieving IBS symptoms due to their potential to alter the microbiome, conclusive scientific evidence remains lacking. This exploratory study aims to assess the bifidogenic properties of four nutritional supplement interventions and their impact on IBS-symptoms, faecal microbiota composition, faecal short-chain fatty acid (SCFA) concentrations, stool pattern, and quality of life (QoL), compared to a placebo control. Seventy subjects with IBS, meeting the ROME IV criteria, participated in this randomised, double-blind, placebo-controlled parallel intervention study. Subjects were assigned to one of the four treatment groups, receiving either resistant starch, pea fibre, chondroitin sulfate, protein hydrolysate, or placebo daily for four weeks. Daily reports on stool pattern and gastrointestinal complaints were collected. Stool samples and questionnaires on dietary intake, symptom severity, QoL, and anxiety and depression were collected at baseline and after the 4-week intervention. The results show no significant increase in *Bifidobacterium* abundance or faecal SCFA levels after the 4-week intervention with any of the four nutritional supplement interventions. While some improvements in symptom severity and QoL were observed within-groups, these were not significantly different from changes observed with placebo. In conclusion, the tested nutritional supplements did not increase *Bifidobacterium* abundance in subjects with IBS within four weeks. Furthermore, we conclude that future studies should consider a run-in period and a larger sample size to study improvements in IBS symptoms.

## Introduction

Irritable Bowel Syndrome (IBS) is a highly prevalent functional gastrointestinal (GI) disorder, with no substantiated clinical treatment and a complicated, diverse and not fully understood, pathophysiology.^(1–3)^ It is known that factors such as dietary patterns, low-grade inflammation, the gut microbiota, and the gut-brain axis play a role in the development of IBS and contribute to the severity of IBS-symptoms.^(4,5)^ People with IBS often report that eliminating dietary triggers, such as spicy and fatty foods, vegetables and cereal-based foods, have the most pronounced beneficial effects on their symptoms.^(5–7)^ However, there is a large variation between individuals in their response to dietary triggers.^(7)^ Dietary restrictions are not recommended for all people with IBS, as this may result in unjustifiable eliminations, potentially leading to nutritional deficiencies and deterioration in overall health.^(8)^ Instead, the use of functional foods can be a powerful strategy to control IBS-symptoms.

A possible mechanism for the improvement in IBS symptoms may involve altering the gut microbiome. Previous cross-sectional trials have shown lower levels of bifidobacteria in subjects with IBS compared to matched controls, regardless of IBS subtype.^(9–13)^ Studies in subjects with IBS using probiotics containing bifidobacteria showed improvements in IBS-symptoms and depression scores, although these results were not consistent.^(14,15)^ In addition to probiotics, the consumption of dietary fibre has been demonstrated to affect gut microbiota composition in some studies, showing a higher abundance of bifidobacteria and increased production of short-chain fatty acids (SCFA).^(16,17)^ Butyrate, one of the most pronounced SCFA and the preferred energy source for colonocytes, is known for its anti-inflammatory properties and positive effects on gut health.^(18)^ However, dietary fibres with different structures may have different effects on the gut microbiota.^(19)^ Prior research investigating different types of resistant starch (types 1 – 4) has demonstrated varying effects on the human gut microbiota. For resistant starch type 4, an increase in bifidobacteria was observed.^(20)^ Animal and *in vitro* studies with insoluble pea fibre have shown an increase in bifidobacteria,^(21,22)^ although this is not shown in human studies yet. Therefore, it is likely that different dietary fibres exert diverse effects on the gut microbiota composition and thereby on the abundance of *Bifidobacterium* and SCFA concentrations, which hold promise for alleviating IBS symptoms.

Another promising nutritional component that is predominantly utilised by the gut microbiota is chondroitin sulphate (CS). In mice, CS has been demonstrated to increase faecal butyrate concentrations and has been associated with ameliorating stress-induced intestinal inflammation.^(23)^ However, limited studies have been performed on the effect of CS on human gut health and the gut microbiota, as it has primarily been investigated as a treatment for joint pain.^(24)^ Additionally, nutritional interventions that have been proven to reduce stress and anxiety may also exert a positive effect on IBS symptoms through modulation of the gut microbiota.^(25)^ Growing clinical and experimental evidence suggests that IBS may result from an alteration in the gut-brain axis.^(4,26)^ Stress and anxiety can impact the gut microbiota composition,^(27,28)^ and play an important role in IBS symptoms by increasing nausea, vomiting, abdominal pain, and altering bowel habits, thereby lowering QoL.^(26)^ Animal studies have demonstrated that the bioactive peptide α-casozepine, a protein hydrolysate derived from bovine αS1-casein, displays anxiolytic-like properties and has proven efficacy in reducing stress by inducing changes in neuronal activity in brain regions involved in anxiety regulation.^(29,30)^ Through this mechanism, α-casozepine may influence the gut-brain axis, potentially impacting gut health. However, it is important to note that these findings from animal models have not been conducted in models of IBS. Moreover, there is evidence suggesting that other protein hydrolysates, may modulate the gut microbiome and promote the growth of bifidobacteria.^(31)^

To summarise, there is a need for functional foods able to alleviate IBS complaints in individuals with IBS. Scientific data is available which suggest that an increase in bifidobacteria, SCFA levels, and a reduction of stress and anxiety may have beneficial effects on individuals with IBS. There are different types of nutritional interventions, with different working mechanisms, that hold promise in alleviating IBS-related complaints by their potential ability to change the gut microbiome and the abundance of *Bifidobacterium.* Despite the abundance of functional foods available on the market as a result of the high market demand by this target group, conclusive scientific evidence supporting the efficacy of these products is often lacking.^(8)^ This exploratory study aims to compare the bifidogenic efficacy of four different nutritional supplements, with different chemical properties, in subjects with IBS. The evaluation encompassed their impact on IBS-symptoms, faecal microbiota composition, faecal SCFA concentrations, stool frequency, stool consistency, and QoL in IBS-subjects, compared to a placebo control.

## Methods & materials

### Ethics

This study was approved by the Medical Ethics Review Committee Utrecht, the Netherlands (NL75824.041.20), and was registered at ClinicalTrials.gov (NCT04790422). The study was conducted in accordance with the principles of the Declaration of Helsinki (Fortaleza, Brazil 2013), and the requirements described in the EU Clinical Trials Directive 2001/20/EC, transposed in the Revision of the Dutch Medical Research involving Human Subjects Act (WMO, effective as of 1 March 2006).

### Study design and procedures

This randomised, double-blind, placebo-controlled intervention study included 70 subjects with IBS, had a total duration of 4 weeks and 5 parallel treatment arms. The primary outcome of this study was the relative abundance of faecal *Bifidobacterium*. Secondary outcomes were microbiota composition, SCFA concentrations, stool frequency, stool consistency, IBS-related complaints, and QoL. Subjects collected faecal samples at baseline and after the 4-week intervention. After collection, the samples were immediately frozen at home, and transported to the research facility on dry ice within 14 days. Subsequently, the faecal samples were stored at –80 ºC until further analysis. Furthermore, daily questionnaires were completed via an EMA app (LifeData, LLC), to report information about their stool frequency (numerical), stool consistency by using the Bristol Stool scale (BSS, scored from 1 to 7, where 1 indicates constipation and 7 indicates diarrhoea), and IBS-related complaints such as bloating, flatulence, and abdominal cramping (rated on a scale of 0 to 10). Additionally, information about supplement compliance and medication intake was reported on a daily basis in this app. Both at baseline and after the 4-week intervention subjects completed a food frequency questionnaire (FFQ), a validated questionnaire to assess symptom severity (IBS-SSS, ranging from 0 to 500; scores <175 denote mild symptoms, 175–300 moderate symptoms, and >300 severe symptoms),^(32,33)^ the validated 34-item Irritable Bowel Syndrome Quality of Life questionnaire (IBS-QOL, ranging from 0 to 100; higher scores indicate a better QoL),^(34,35)^ and the validated Hospital Anxiety and Depression Score assessment (HADS, with scores ranging from 0 to 21 for both anxiety and depression; scores ≥ 8 suggest symptoms of anxiety or depression).^(36)^

### Study subjects

Subjects were recruited in May 2021. The study was performed from June 2021 to July 2021 in the Netherlands and was conducted in a corona-proof setting, completely at home and online. Subjects were recruited using the Wageningen University & Research subject database, by advertisements on social media such as Facebook and Instagram, and via the website and social media of the Dutch IBS patient association. An online information meeting was organised prior to the start of the study to explain all study procedures and measurements. All subjects willing to participate in the study signed the informed consent form. Inclusion criteria were: IBS subjects meeting the Rome IV criteria, age between 18 and 65 years, and BMI between 18.5 and 30 kg/m^2^. The main exclusion criteria were: 1) having gastrointestinal or other relevant diseases that could affect study results, 2) a history of intestinal surgery, 3) antibiotic treatment within the 3 months before the study, 4) use of medication or irregular use of nutritional supplements, such as prebiotics and probiotics, that could interfere with the study outcomes, or 5) following a FODMAP-restricted diet. Eligibility was assessed using an online screening questionnaire. Subjects were enrolled by the study coordinator.

### Nutritional intervention products

The four tested nutritional supplements were Resistant starch type 4 (RS, NOVELOSE®3490, Ingredion, USA), Pea Inner Fibre (PIF, I50M, Roquette, France), Chondroitin Sulphate (CS, Bioiberica S.A.U., Spain), and Protein hydrolysate cow milk (PHCM, an αS1-casein hydrolysate, containing α-casozepine, Ingredia, France). The placebo supplement consisted of Maltodextrin (GLUCIDEX® IT 19 P, Roquette, France). Each of these supplements was consumed twice daily, one sachet in the morning and one sachet in the evening taken together with two spoons of banana compote (Frulla, Italy). For all supplements, the most effective and regularly consumed dosage was chosen based on literature and information provided by the providers of the supplements (Table 1). PIF and RS are insoluble fibres, with study dosages set at 20 g/d for both RS and PIF (PIF consists of 10 g fibre, the other 10 g consists of starch and protein). The CS, a soluble fibre supplement, was given in a daily dose of 1.2 g and the PHCM in a daily dose of 300 mg. Both CS and PHCM supplements were mixed with Maltodextrin to make supplements of 10 grams per sachet. The second dose of 10 grams, which was consumed in the evening, consisted of the placebo supplement for the CS and PHMC arms. Because a sudden increase in fibre might lead to GI complaints, subjects in all arms had a five-day run-in period where they consumed only 1 sachet in the morning, followed by both sachets for the remainder of the four weeks. All sachets were uniformly packed, to ensure similarity of the interventions.

**Table 1.** Overview of supplement dosages per day

Supplement	Dosage /day
Resistant starch type 4, NOVELOSE®3490 (RS)	20 g
Pea Inner Fibre 150M (PIF)	20 g
Chondroitin Sulphate (CS)	1.2 g (added with maltodextrin to 10 g) + 10 g placebo
Protein hydrolysate cow milk, an αS1-casein hydrolysate, containing α-casozepine (PHMC)	300 mg (added with maltodextrin to 10 g) + 10 g placebo
Maltodextrin, GLUCIDEX® IT 19 P (Placebo control)	20 g

### Sample size and randomisation

No data on the effect size of the intervention products tested in the current study were available. Therefore, the sample size calculation was based on data from a previous study investigating the effect of 3.5 gram Galactooligosaccharides (GOS) on the relative abundance of *Bifidobacterium* in individuals with IBS (relative abundance; mean difference (delta) = 2.26, SD of the difference = 0.67).^(37)^ To account for the possibility of a smaller effect in our study, we assumed approximately half this effect size for our calculation, resulting in a mean difference of 1.1, with a variance of 0.6. Based on a power of 80% and a significance level of 0.05, 12 subjects per treatment arm would be needed to demonstrate a significant difference. To compensate for potential drop-outs, 14 subjects per treatment arm were included (70 subjects in total, divided across 5 arms).

Subjects were stratified according to age (2 groups) and BMI (2 groups) and randomly assigned to one of the five treatment arms using block randomisation by the study coordinator. Blocks of five subjects were created, and the allocation sequence within each block was generated using random numbers in Excel. An independent researcher assigned a unique code to each of the study products. All researchers of the project team, with the exception of the independent researcher, were kept blind to the assignment of treatment, and so were the study subjects. After database lock and signing of the statistical analysis plan, the key to the treatment allocation was shared with the other researchers.

### Microbiota analysis

DNA was isolated from 0.25 gram of wet faeces using a previously published protocol.^(38)^ Isolated and purified DNA was used for PCR amplification of the V4 region of 16S rRNA gene with barcoded primers 515F (5’-GTGY CAGC MGCC GCGG TAA-3’)^(39)^ and 806R (5’-GGAC TACN VGGG TWTC TAAT-3’)^(40)^ in duplicate. PCR was performed in 50 µl reaction mix containing 10 µl 5x HF buffer (Thermo Fisher Scientific, Vilnius, Lithuania), 1 µl dNTPs (10 mM, Thermo Fisher Scientific), 0.5 µl Phusion Hot start II DNA polymerase (2 U/µl, Thermo Fisher Scientific), 36.5 µl nuclease-free water (Promega, Madison, WI, USA), 1 µl DNA template (20 ng/µl), and 1 µl sample-specific barcoded primer (10 µM).^(41)^ PCR was performed as follows: 98 ℃ for 30 s, followed by 25 cycles of 98 ℃ for 10 s, 50 ℃ for 10 s, 72 ℃ for 10 s, with a final extension of 7 min at 72 ℃.^(41)^

The PCR product was purified with the CleanPCR kit (CleanNA, The Netherlands), and quantified using the Qubit dsDNA BR Assay kit (Invitrogen by Thermo Fisher Scientific, Eugene, OR, USA). An equimolar mix of purified PCR products was prepared and sent to Novogene (Cambridge, United Kingdom) for sequencing. Raw sequencing data were processed with NG-Tax pipeline 2.0 with default settings.^(41,42)^ Amplicon Sequencing Variant (ASV) picking and taxonomic assignments were performed using the SILVA 138.1 database. Raw sequencing data are available at the European Nucleotide Archive under accession number PRJEB56219.

### Short-chain fatty acid analysis

The concentrations of acetate, propionate, and butyrate were measured in faecal water, using High-Performance Liquid Chromatography (HPLC, Shimadzu Prominence-I LC2030C-Plus, Shimadzu, Duisburg, Germany) equipped with a Shoedex SH1821 column (Showa Denko, New York, USA) and RID-20A refractive index detector (Shimadzu).

To prepare the sample, 0.2 grams of wet faeces was mixed with 0.8 ml of distilled water, and subsequently centrifuged at 21130 RCF for 5 min at 4°C. The supernatant went through a pre-treatment step with Carrez reagents (A and B) to deproteinise the samples. The Carrez A solution consisted of 0.1 M K_4_Fe(CN)6·3H_2_O and the Carrez B solution consisted of 0.2 M ZnSO_4_·7H_2_O. Both solutions were stored at 4°C. Next, 500 µl of supernatant was mixed with 250 µl cold Carrez A solution, followed by mixing with 250 µl cold Carrez B solution. Afterwards, the sample was centrifuged at 15000 RCF for 5 min at 4°C, and the clear supernatant was collected for measurement. The HPLC conditions included eluent composition of 0.01 N H_2_SO_4_, eluent flow rate set at 1 ml/min, column oven temperature maintained at 54°C, flow rate of 0.8 ml/min, and utilisation of 10 mM DMSO as the internal standard. All data were processed by the Chromeleon software (version 7.2.9, Thermo Fisher Scientific).

### Data and statistical analyses

**Figure 1.**
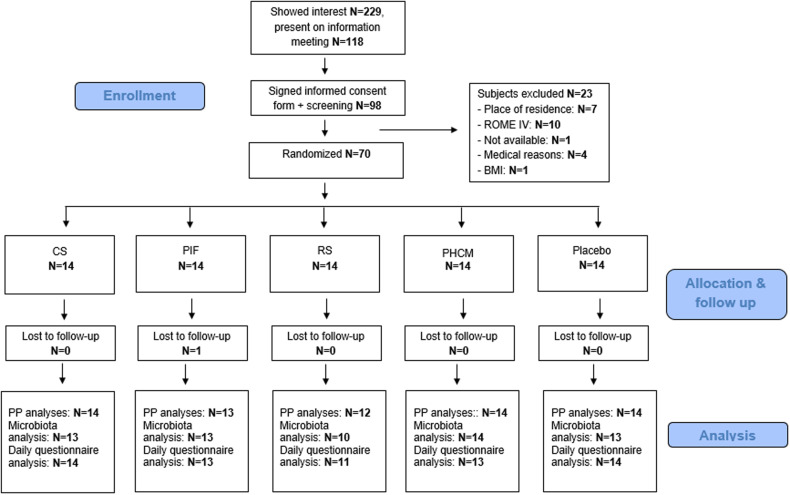
Study participant Flow-chart. Flowchart of study subjects from recruitment and screening to final per protocol (PP) data analyses.

Intention-to-treat (ITT) analyses were performed for all outcomes and for all randomised subjects who received any study product and who completed all four weeks of the intervention period (see Fig. 1). Per protocol (PP) analyses excluded subjects with major protocol deviations related to medication intake or illnesses, that interfered with the study outcome, or with a low questionnaire compliance or intervention product compliance. Intervention product compliance was reported by subjects themselves, using the daily questionnaire in the app.

Continuous data are presented as mean ± standard deviation if normally distributed or median [interquartile range] when skewed. All questionnaire data were analysed with R (version 1.3.959), microbiota data were analysed using R (version 4.2.2). All statistical analyses were performed on blinded data, except for the placebo group.

16S rRNA gene read count data were transformed to microbial relative abundance, as implemented in the Microbiome R package.^(43)^ To measure the change in relative abundance of *Bifidobacterium* over time, fold changes were calculated. To determine differences in relative abundance of *Bifidobacterium* over time between the placebo and treatment groups, a paired sample T-test was used when the data was normally distributed, and the paired Wilcoxon test was used when the data were not normally distributed.

Microbial diversity indices including Inverse Simpson (richness and evenness of species in a community) and Faith’s Phylogenetic Diversity (phylogenetically weighted measure of richness) were calculated at ASV level, as implemented in the Phyloseq^(44)^ and Picante^(45)^ R packages, respectively. Since these data were not parametric, (un)paired Wilcoxon test was conducted to determine the difference between groups. Pairwise weighted Unifrac^(46)^ and unweighted UniFrac^(47)^ distance-based principal coordinates analysis (PCoA) were used to visualise microbial community variation at the ASV level. Permutational multivariate analysis of variance (PERMANOVA) was used to test for significant differences between groups, as implemented in the Vegan R package.^(48)^ Differences in microbiota data between week 0 and week 4 were compared with the paired sample T-test when normally distributed or the paired Wilcoxon test when not normally distributed. RDA analysis was performed with the Vegan package. All figures were generated using the ggplot2 package.^(49)^

Unpaired T-test was used to compare the post-intervention differences for faecal SCFA concentration data between placebo and treatment groups. Unpaired T-test was also used to compare differences between each treatment group and the placebo group as implemented in the rstatix R package.^(50)^

Stool frequency, stool consistency, and IBS-related complaints (abdominal pain, bloating, and flatulence) were reported daily. For stool consistency, a daily mode per individual was calculated. Differences over time between the treatment groups and placebo (Time/Intervention interaction) were assessed using linear mixed models for repeated measures with subject as random effect. Questionnaire data on symptom severity, QoL, and HADS, reported at baseline and after the 4-week intervention, were analysed using linear mixed models, with time and treatment as main effects and subject as random effect. Interaction effects were also examined. Within-group effects for questionnaire data were analysed using paired sample T-test. To test for changes in fibre intake over time, delta values were calculated and analysed using one-way ANOVA.

False discovery rate (FDR) correction based on Benjamini–Hochberg procedure was used to correct for multiple testing, when applicable for microbiota and SCFA markers. In all cases, (adjusted) P-values < 0.05 were considered statistically significant, and adjusted P-values between ≥ 0.05 and < 0.1 were considered a trend.

## Results

### Participants and baseline characteristics

A total of 70 subjects with IBS were included in this study, of whom 69 completed the 4-week study period (58 females and 11 males). Baseline characteristics of the study subjects per treatment group are shown in Table 2. The mean age of all study subjects was 37.8 years (range 18–62 years) with an average BMI of 23.5 kg/m^2^ (range 18.6–29.7 kg/m^2^).

**Table 2.** Study participant baseline characteristics (ITT population, n = 69)

	CS N = 14	PIF N = 13	RS N = 14	PHCM N = 14	Placebo N = 14	Total N = 69
Age in years^a^	41.1 (16.3)	33.6 (11.3)	39.1 (14.3)	36.4 (9.9)	38.3 (11.6)	37.8 (13.1)
Sex in counts n total (female)	14 (11)	13 (11)	14 (13)	14 (11)	14 (12)	69 (58)
BMI in kg/m^2^ ^a^	23.4 (2.3)	23.3 (3.1)	23.8 (2.8)	23.3 (2.9)	23.8 (3.3)	23.5 (2.9)
Stool frequency per day^a^	1.7 (0.8)	1.6 (0.8)	1.7 (0.8)	1.9 (0.6)	1.4 (0.3)	1.6 (0.7)
Stool consistency (BSS mode per day)^a^	3.9 (1.4)	3.6 (1.4)	4.0 (1.3)	4.7 (0.8)	4.4 (0.8)	4.1 (1.2)
ROME sub group						
IBS-C, n (%)	5 (36%)	2 (15%)	4 (28%)	2 (14%)	2 (14%)	15 (22%)
IBS-D, n (%)	5 (36%)	4 (31%)	2 (14%)	6 (43%)	7 (50%)	24 (35%)
IBS-M, n (%)	4 (28%)	6 (46%)	8 (57%)	6 (43%)	5 (36%)	29 (42%)
No abnormal stools, n (%)		1 (8%)				1 (1.4%)

CS, Chondroitin Sulphate, Bioiberica S.A.U., Spain; PIF, Pea Inner Fibre 150M, Roquette, France; RS, Resistant Starch, Ingredion, USA; PHCM, Protein Hydrolysate Cow Milk, Ingredia, France; BMI, Body Mass Index; IBS-C, IBS predominant constipation; IBS-D; IBS predominant diarrhoea; IBS-M, IBS mixed subtype; BSS, Bristol Stool Score.

a Mean (SD, Standard Deviation).

After inclusion, one subject dropped out of the study due to worsening of GI complaints. Therefore, 69 subjects completed the study (Fig. 1). For the PP analyses, two subjects were excluded from all analyses because of medical reasons and supplement compliance <75%. For the microbiota analysis, four additional subjects were excluded from the PP analyses because of a missing sample (n = 1), antibiotic use (n = 1), and low number of reads (n = 2). Also, for the daily questionnaires, two extra subjects were excluded from the PP analyses due to a daily questionnaire compliance of <70%.

### Subject compliance, adverse events, and food intake

Compliance to the intake of study supplements was high. Based on self-reported data in the daily app, on average, subjects consumed 98.1% of all sachets. Additionally, the compliance to completion of the daily questionnaires was high. Overall, 95.6% of all questionnaires were completed by the subjects.

A total of four adverse events, possibly or probably related to the treatment, or study procedures, were reported. All these events were mild to moderate and involved GI complaints, such as abdominal pain and diarrhoea. No serious adverse events were reported during the study.

For all treatment groups, fibre and energy intake decreased after the 4-week intervention compared to baseline, based on dietary intake data excluding supplement use. In the CS treatment group, this reduction was statistically significant compared to baseline, with fibre intake decreasing by Δ = –3.37 g (SD = 3.46 g, P < 0.01) and energy intake decreasing by Δ = –355.7 kcal (SD = 359.5 kcal, P < 0.01). Additionally, compared to the placebo group, the reduction in the CS group was also significant (placebo group: fibre intake Δ = 2.5 g (SD = 5.03 g), energy intake Δ = –44.0 kcal (SD = 243.5 kcal), comparison placebo with CS group: P = 0.01).

### Faecal microbiota composition

**Figure 2.**
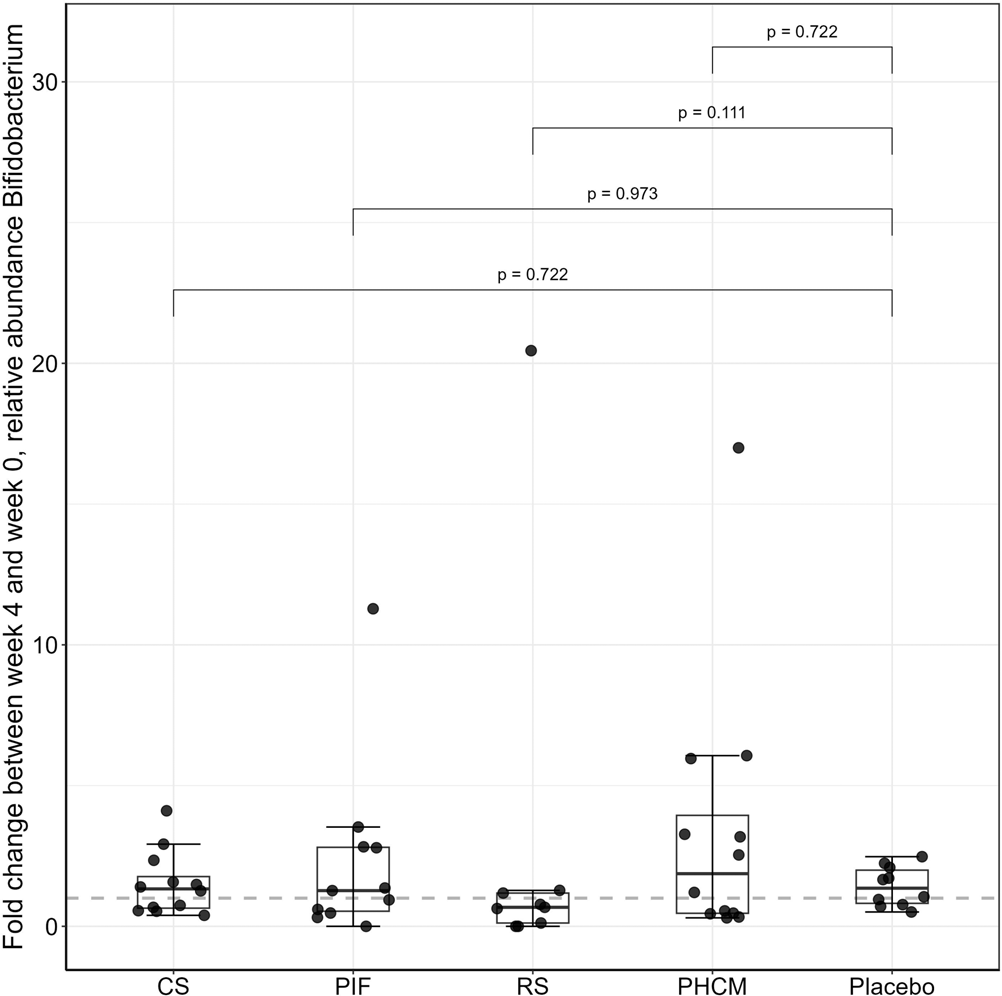
Fold change of the relative abundance of *Bifidobacterium* between week 4 (after supplementation) and week 0 (before supplementation) for CS (n = 13), PIF (n = 13), RS (n = 10), PHCM (n = 14) and placebo (n = 13).

Four-week supplementation with CS, PIF, RS, or PHCM did not increase the relative abundance of *Bifidobacterium* compared to placebo (Fig. 2 and Supplementary material Table S1). For all groups, the relative abundance of *Bifidobacterium* after the 4-week supplementation did not differ compared to baseline (Supplementary material, Figure S1).

The change in faecal microbiota alpha diversity was not significantly different after the 4-week supplementation with either CS, PIF, RS, or PHCM compared to placebo (Supplementary material, Figure S2). For all supplements, the faecal microbiota alpha diversity after the 4-week supplementation was not significantly different compared to baseline (Supplementary material, Figure S3).

Microbial community structure (beta diversity) did not change after the 4-weeks supplementation, for any of the supplements (Supplementary material, Figure S4). At baseline, however, the unweighted UniFrac parameter, which solely takes into account the absence or presence of bacteria, did show a significant difference between groups (P = 0.001). Since the weighted UniFrac distance, which also accounts for the abundance of the taxa, did not reveal significant differences between the groups at baseline, this suggests that microbial differences between the groups at baseline were related to absence/presence of low-abundant taxa.

### Faecal short-chain fatty acid concentration

There were no significant differences observed in the changes of SCFA concentrations, including total SCFA, acetate, propionate, and butyrate, following the 4-week supplementation period with CS, PIF, RS, or PHCM when compared to placebo. For all groups, SCFA concentrations after the 4-week supplementation did not differ from baseline (Supplementary material, Figure S5).

### IBS symptoms and quality of life

**Figure 3.**
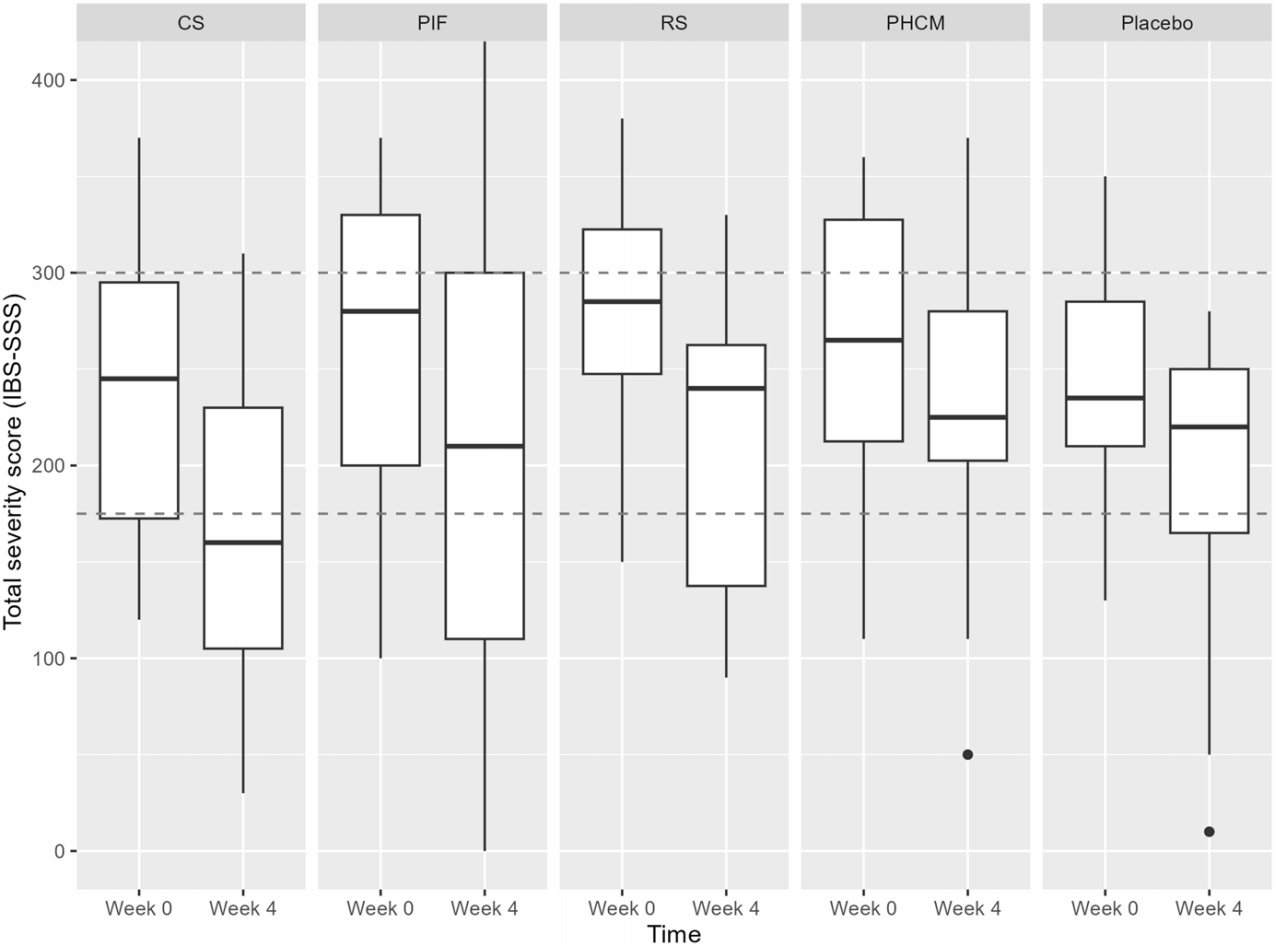
Boxplot depicting IBS symptom severity scores (IBS-SSS) at week 0 (before supplementation) and week 4 (after supplementation) across the different treatment groups: CS (n = 14), PIF (n = 13), RS (n = 12), PHCM (n = 14) and placebo (n = 14). IBS-SSS scores above 300 are considered severe and scores below 175 are considered mild. The boxplots represent the interquartile range (IQR), with the median marked by the line inside the box. Whiskers extend to the minimum and maximum scores within 1.5 times the IQR, while outliers are plotted individually with dots.

Overall, IBS-SSS scores ranged from 100 to 380 at baseline and between 0 and 420 after the intervention (scale: 0–500). On average, subjects in all treatment groups had moderate IBS severity symptoms (scores between 175 and 300). IBS-SSS scores improved significantly after the 4-week supplementation with CS (Δ = –66 ± 102, P = 0.03, paired sample T-test) and RS (Δ = –72 ± 102, P = 0.03, paired sample T-test), compared to baseline values. Importantly, this was also observed after the 4-week supplementation with placebo (Δ = –46 ± 70, P = 0.03, paired sample T-test). The change in IBS-SSS score after the 4-week supplementation with CS and RS was not significantly different compared to placebo, based on linear mixed model analysis (Fig. 3). No difference in IBS-SSS scores was found after the 4-week supplementation with PIF and PHCM.

Subjects with an improved IBS-SSS score of ≥ 50 points were classified as responders, as described previously.^(32)^ There was no significant difference in the response rate observed between the treatment groups and the placebo group.

**Figure 4.**
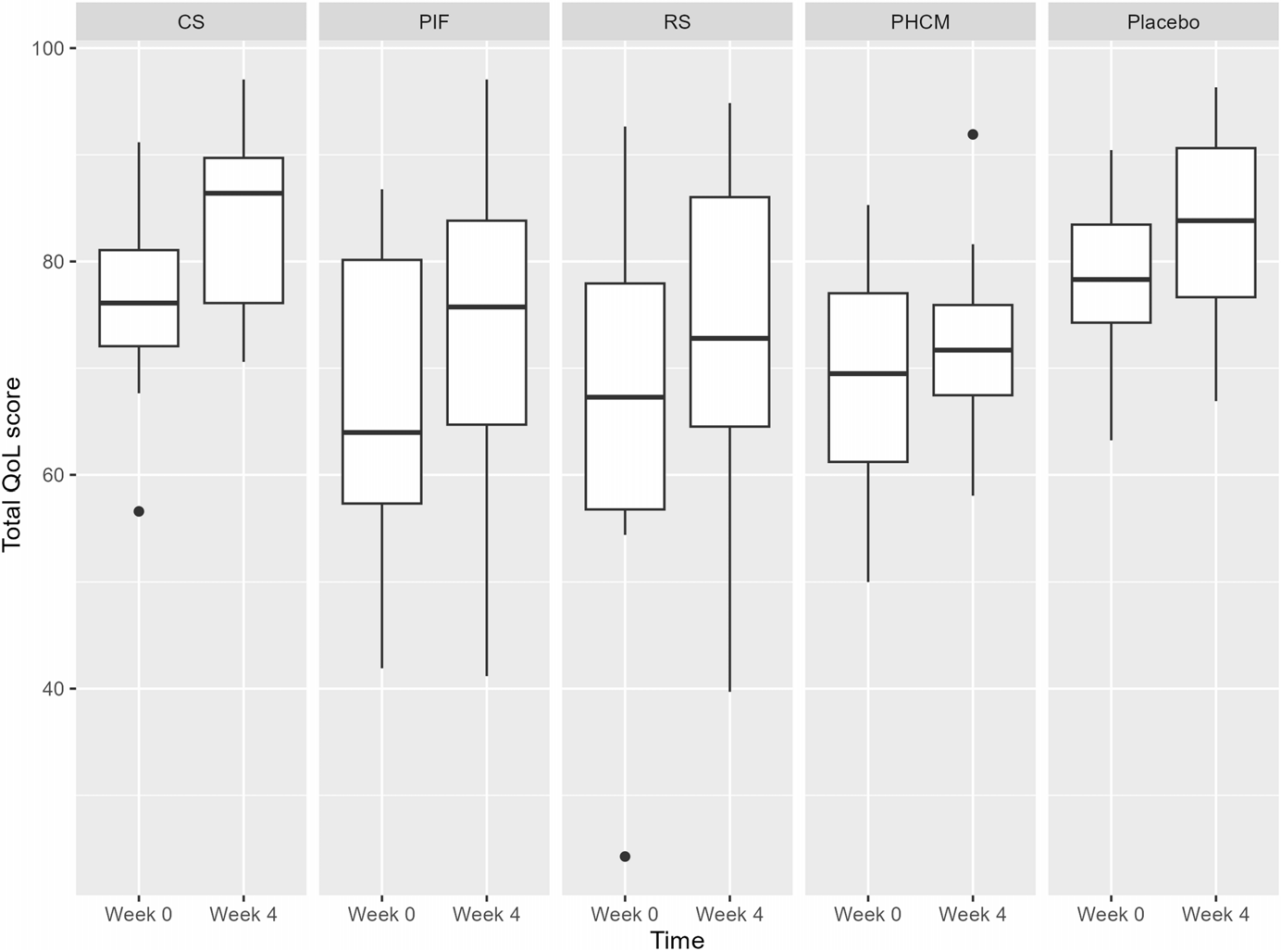
Quality of life (QoL) scores at week 0 (before supplementation) and week 4 (after supplementation) across different treatment groups: CS (n = 14), PIF (n = 13), RS (n = 12), PHCM (n = 14) and placebo (n = 14). The boxplots represent the interquartile range (IQR), with the median marked by the line inside the box. Whiskers extend to the minimum and maximum scores within 1.5 times the IQR, while outliers are plotted individually with dots.

Examining the group as a whole, QoL average scores ranged from 24 to 93 at baseline and from 40 to 97 after the intervention (on a 0–100 scale), with higher scores indicating a better quality of life. A significant improvement in QoL scores was observed after the 4-week supplementation with CS (Δ = 8.0 ± 7.8, P = 0.002, paired sample T-test) compared to baseline (Fig. 4). The same effect was observed after the 4-week placebo supplementation (Δ = 4.3 ± 6.2, P = 0.002, paired sample T-test). The improvements in QoL scores for the different treatment groups were not significantly higher compared to placebo, based on linear mixed model analysis.

Average anxiety scores ranged from 5.5 to 6.9 for subjects in the placebo group, while for all other treatment groups they ranged from 7.4 to 8.2. These findings suggest that the scores of the treatment groups were close to the threshold for anxiety symptoms (scores ≥ 8). Depression scores were on average between 2.8 and 4.8 for all treatment groups, including the placebo, indicating that subjects in general did not have depressive symptoms (scores < 8). Anxiety and depression scores did not significantly change after the 4-week supplementation period for all groups. Changes in depression and anxiety scores were not significantly different between the treatment groups and the placebo (data not shown).

In general, abdominal pain, bloating and flatulence symptoms were low during the study. Average GI symptom scores ranged from 2 to 4 on a 10-point scale, with higher scores indicating more GI symptoms. GI symptoms did not significantly change over time within treatment groups. Although for bloating, scores increased significantly after the 4-week CS supplementation compared to placebo (P = 0.02, based on linear mixed model analysis, data not shown).

### Stool pattern

Throughout the study, there were no significant alterations in stool frequency and stool consistency over time within any of the groups (data not shown). Additionally, there were no statistically significant differences observed between the treatment groups and the placebo group.

## Discussion

This double-blind randomised placebo-controlled parallel study included 70 subjects with IBS, including all sub-types (IBS-C, IBS-D, IBS-M, and IBS-U). The study aimed to investigate the effects of four weeks supplementation with four different functional food ingredients on the relative abundance of faecal *Bifidobacterium*, total faecal SCFA concentrations, IBS severity, QoL, anxiety, depression, IBS-related GI complaints, and stool pattern, as compared to placebo. Our results showed no significant difference in the relative abundance of faecal *Bifidobacterium* and SCFA concentrations after the 4-week supplementation with these supplements. For the subjective outcome measures, a few within-group effects were observed, although these were not significantly different from the placebo.

In line with daily practice, in which people with IBS often use a variety of supplements, four potentially effective nutritional supplements were selected for this study. All supplements had different hypothesised physiological mechanisms of action, with PIF and RS linked to bifidogenic effects, CS potentially enhancing butyrate production and thereby altering microbiota composition, and PHMC expected to impact the gut-brain axis.^(21,22,24,51,52)^ Although all four supplements are acknowledged for their effects on the microbiome and/or gut-brain axis in existing literature, primarily in animal studies, these effects were not confirmed in the current study.

The expected benefits of nutritional supplements with potential bifidogenic properties for individuals with IBS were based on prior research, indicating a reduced abundance of *Bifidobacterium* in individuals with IBS compared to healthy controls.^(9–13)^ However, an increase in relative abundance of *Bifidobacterium* was not always associated with relief of symptoms, suggesting that the role of the microbiome is complex and largely not unidirectional.^(13,53)^ Given the absence of a healthy control group in our study, we are unable to confirm whether the baseline levels of *Bifidobacterium* in our study population were indeed lower compared to healthy controls. Wang, T. *et al.* (2022) studied the longitudinal dynamics of faecal microbiota in subjects with IBS compared to healthy controls, using comparable procedures and subjects from the same geographical location as our study population. At baseline, the relative abundance of *Bifidobacterium* was significantly lower in their IBS population compared to our study population, which was more similar to their healthy control group.^(13)^ This difference in baseline microbiota composition can be attributed to inter-individual and/or study population differences,^(54)^ and various parameters that can affect microbiome composition – for instance differences in dietary intake, IBS severity, subtype and symptoms, seasonal effects, age, and BMI.^(13,55,56)^ Additionally, there is no conclusive consensus about the reduced levels of bifidobacteria within the IBS population.^(57)^ Nevertheless, these relatively high baseline levels of *Bifidobacterium* might have left less room for improvement in our study population.

Besides the relatively high initial abundance of *Bifidobacterium*, the overall initial microbiota composition of subjects at baseline might have impacted the response to the different nutritional interventions. Due to inter-individual variations in microbiota profiles, microbes involved in supplement degradation will differ between individuals, either through direct action of the microbes or via cross-feeding, leading to the production of diverse metabolites.^(51,56)^ This variation in initial microbiota profiles could have contributed to differentiation between individuals who responded and those who did not respond to the interventions. This variance may have reduced the overall treatment effects.

### Strengths and limitations

It is widely acknowledged that people with IBS exhibit a high susceptibility to placebo effects, alongside considerable variability in subjective complaints within and across individuals.^(58)^ For this reason, the inclusion of a parallel placebo group makes the design of this study more robust. Since all 70 subjects were recruited and included during the same period (May 2021 – July 2021), this might have reduced the impact of external factors, that might otherwise have contributed to the inter-individual variability. Moreover, the number of drop-outs was low, and adherence to study procedures was high, as monitored on a daily basis. This might be attributed to the daily reminders sent via the Lifedata app, and the easy accessibility of the study team by phone and e-mail, seven days a week.

Despite the study being well executed, none of the nutritional supplements demonstrated any significant impact on our outcomes. This lack of effectiveness, compared to placebo, may be attributed to the limited efficacy of the supplements. However, other contributing factors that might have prevented significant results could include the relatively high initial abundance of *Bifidobacterium*, the variation in initial microbiome profiles, natural variation in symptoms, especially in different IBS subtypes (IBS-C, IBS-D, IBS-M and IBS-U), and placebo effects that might have hampered results, as described above. Although our study was adequately powered to detect differences in the relative abundance of *Bifidobacterium*, this effect was not observed. Future studies should consider larger sample sizes to assess effects on more subjective outcomes, perhaps stratify for IBS subtype, and include a run-in period of at least 2 weeks. The latter is known to reduce placebo response rates.^(58)^ Inclusion of a positive control, such as a prebiotic GOS supplement, alongside the placebo control group, could also help to account for these external influences. Additionally, higher supplement dosages might be needed to show efficacy.^(52)^

### Conclusion

We conclude that the nutritional supplements, at the dosages tested in the current study, were not able to increase the relative abundance of Bifidobacterium in subjects with IBS within a 4-week intervention. Some within-group effects were observed on subjective IBS-related outcomes, although these were not significantly different from placebo. To account for external factors affecting variation in outcomes and high placebo response rates, we recommend future studies to include a run-in period of at least 2 weeks, increase the sample size, and include a positive control alongside a placebo control group, particularly for studying subjective outcomes related to IBS-related symptoms.

## Supporting information

van den Belt et al. supplementary material 1 van den Belt et al. supplementary material

van den Belt et al. supplementary material 2 van den Belt et al. supplementary material

## Supplementary material

The supplementary material for this article can be found at https://doi.org/10.1017/jns.2025.10021

## Data sharing

Raw 16S rRNA gene sequence data have been deposited in the European Nucleotide Archive (http://www.ebi.ac.uk/ena) under accession number PRJEB56219. The R code for the statistical analysis used in this study is available at a public repository on GitLab (https://git.wur.nl/afsg-microbiology/publication-supplementary-materials/2023-liu-ibs_clinical_comparison_study).
