# Supplementary material for: Randomised controlled trial: nutritional supplements to relieve irritable bowel syndrome symptoms by targeting the gut microbiota
Source: J Nutr Sci. 2025 Jul 11;14:e46. doi: 10.1017/jns.2025.10021 (PMC12278178; doi:10.1017/jns.2025.10021)
Supplement: van den Belt et al. supplementary material 1 — van den Belt et al. supplementary material [file S2048679025100219sup001.docx]

**Supplementary data**

**
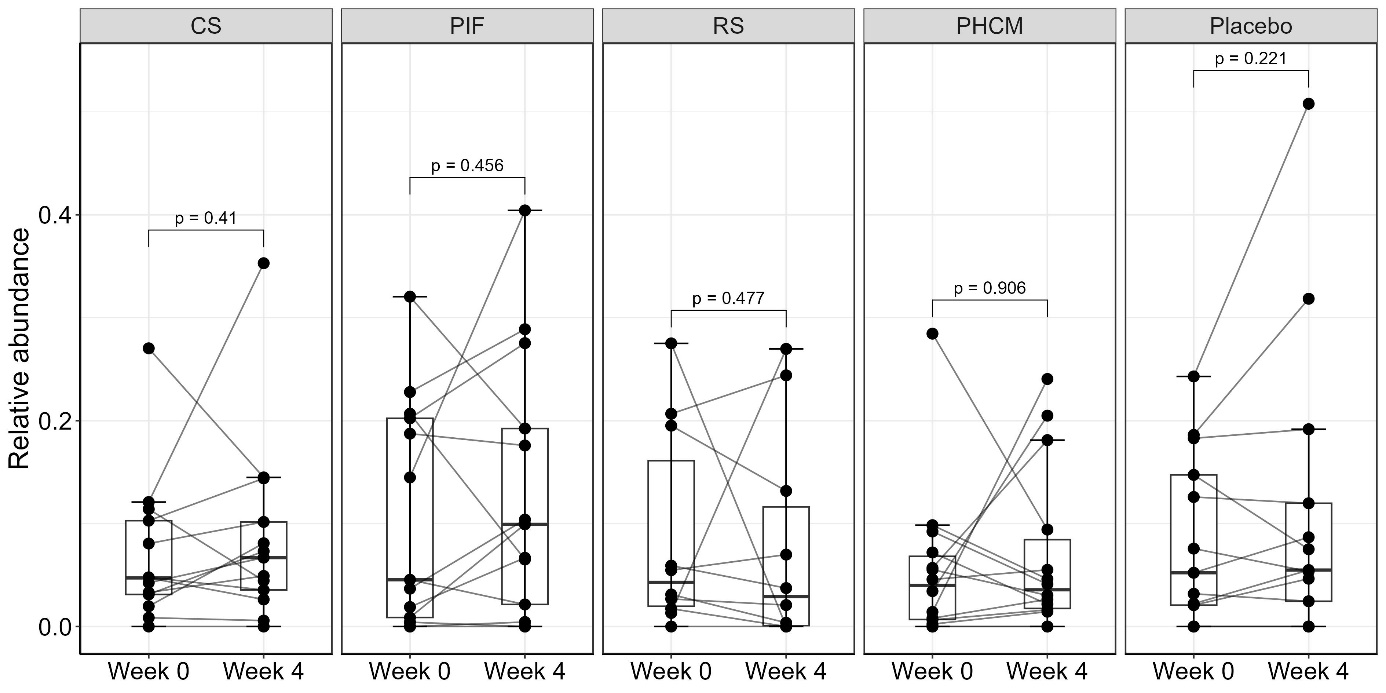
**

**Figure S1.** Relative abundance Bifidobacterium before and after 4-week supplementation with CS, PIF, RS, PHMC or placebo.

**Table S1. Relative abundance Bifidobacterium before and after 4-week supplementation with CS (n=13), PIF (n=13), RS (n=10), PHMC (n=14) or placebo (n=13).**

| Supplement | Mean / SD | Week 0 | Week 4 | Delta |
| --- | --- | --- | --- | --- |
| CS | mean | 7.1% | 8.7% | 1.6% |
|  | sd | 7.2% | 9.2% | 8.2% |
| PIF | mean | 10.8% | 13.1% | 2.3% |
|  | sd | 11.1% | 12.9% | 10.1% |
| RS | mean | 8.8% | 7.8% | -1% |
|  | sd | 9.9% | 10.3% | 12.8% |
| PHCM | mean | 5.5% | 6.9% | 1.4% |
|  | sd | 7.4% | 8% | 10.3% |
| Placebo | mean | 8.4% | 11.4% | 3% |
|  | sd | 8.4% | 14.8% | 8.4% |

*Table shows the relative abundance of* Bifidobacteria *in stool samples, expressed as percentages (% of total bacterial composition). Mean and standard deviation (SD) are reported per group and per timepoint (Week 0, Week 4). Delta indicates the change from Week 0 to Week 4.*


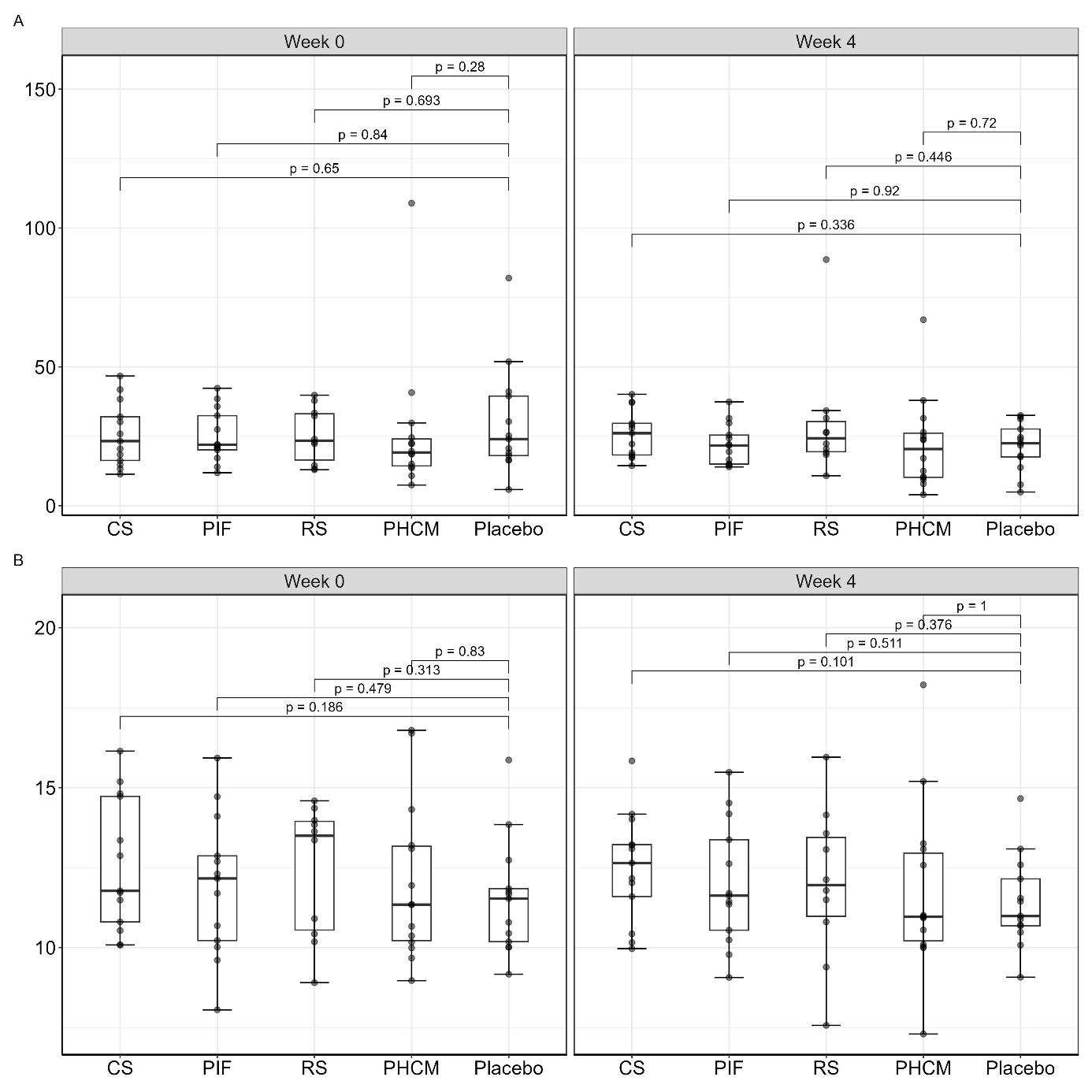


**Figure S2.** **Faecal microbial alpha diversity** indicated with the inverse Simpson (A) measure, indicating the evenness and richness of species, and with the Phylogenetic Diversity (B) measure, indicating the diversity across a phylogenetic tree of species. Data shows the microbial diversity before (week 0) and after (week 4) supplementation with either CS, PIF, RS, PHCM or placebo.


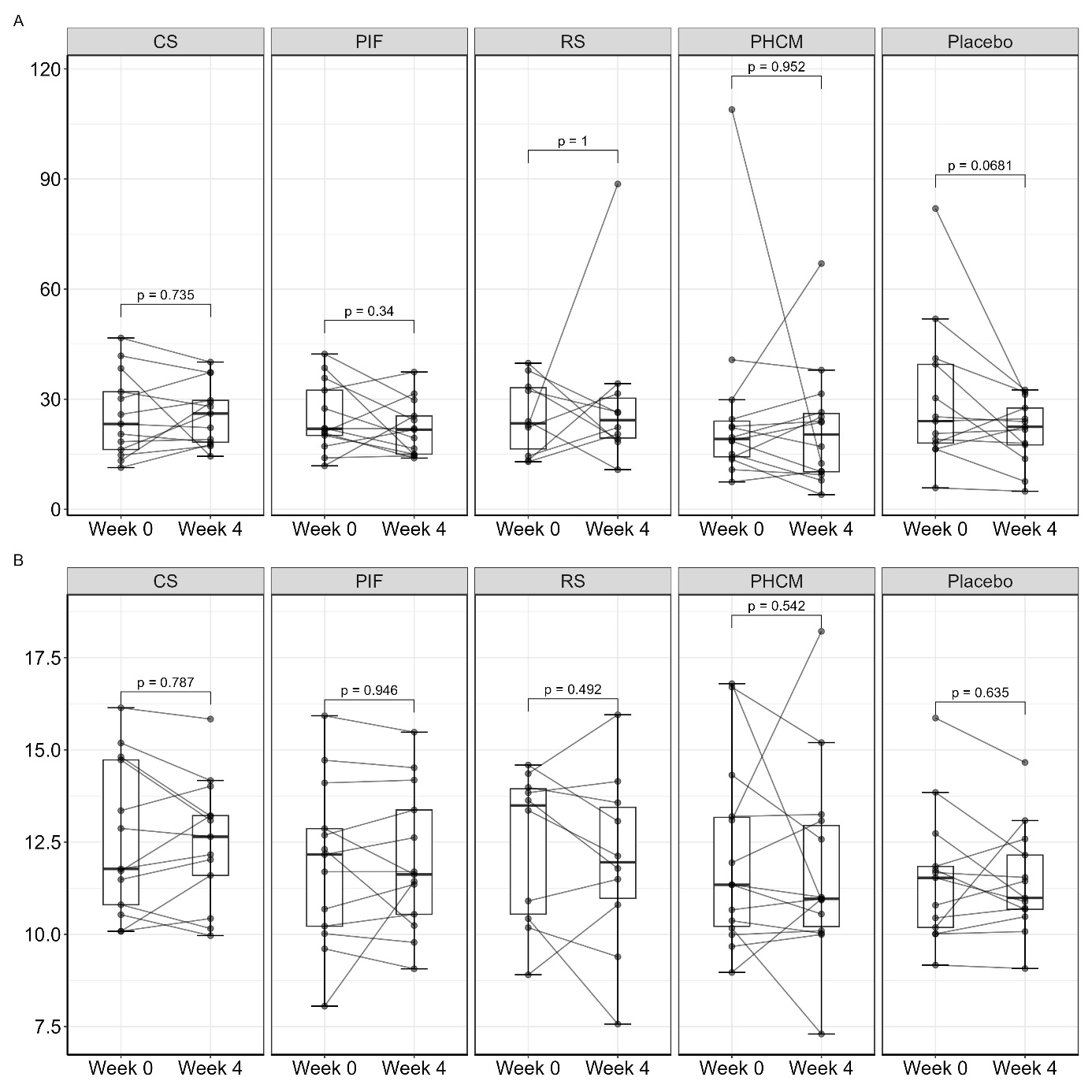


**Figure S3.** Microbial alpha diversity index for richness and evenness (A; Inverse Simpson) and diversity across trees (B; Phylogenetic Diversity) before and after 4-week supplementation with CS, PIF, RS, PHMC or placebo.


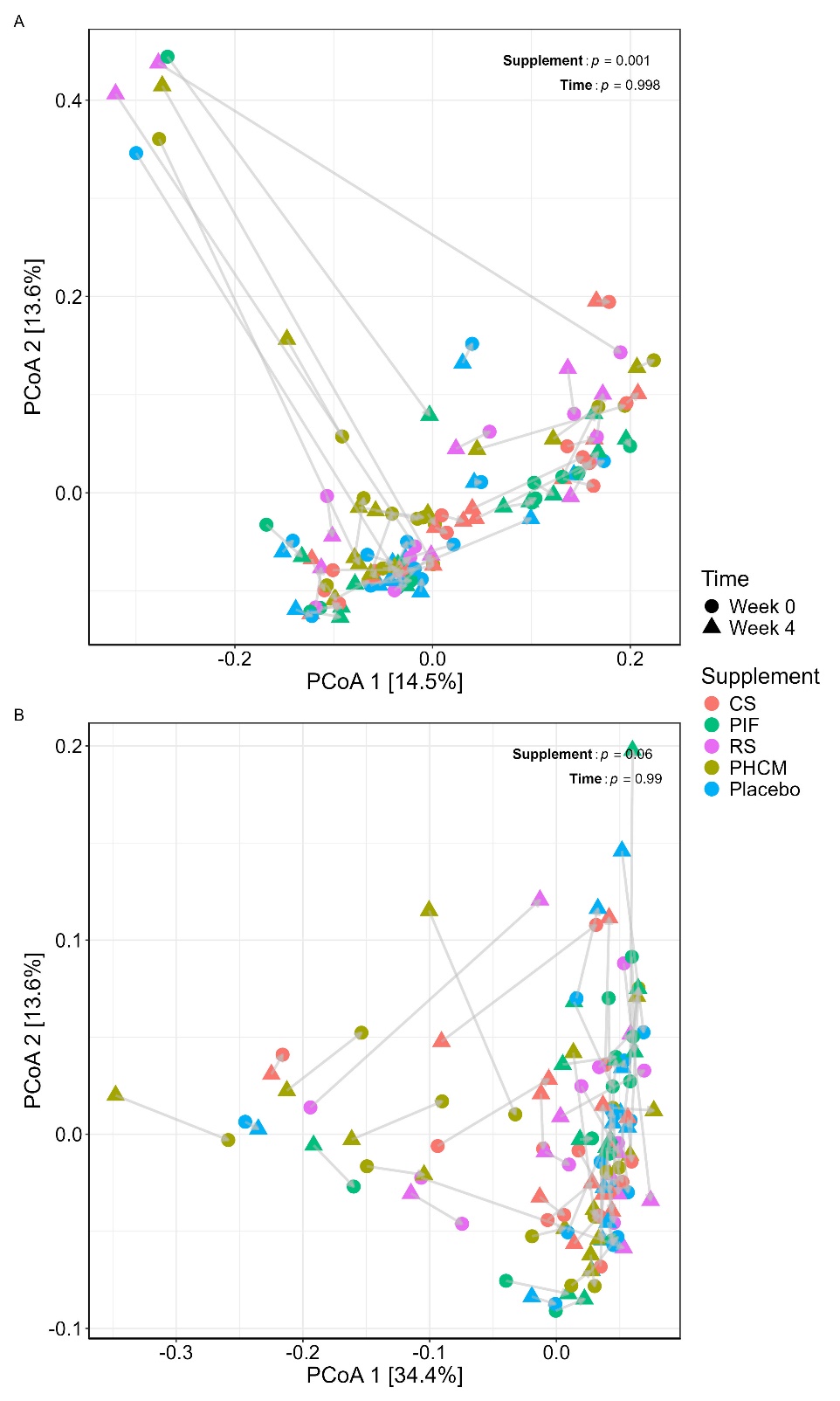


**Figure S4. Comparison of structure of microbiota community (beta-diversity) over time**Data shows the comparison between week 4 and week 0, between each treatment group (CS, PIF, RS, PHCM and placebo) based on unweighted UniFrac (A) and weighted UniFrac (B) pairwise distance using ASV level data. Level of significance between groups was tested by PERMANOVA.


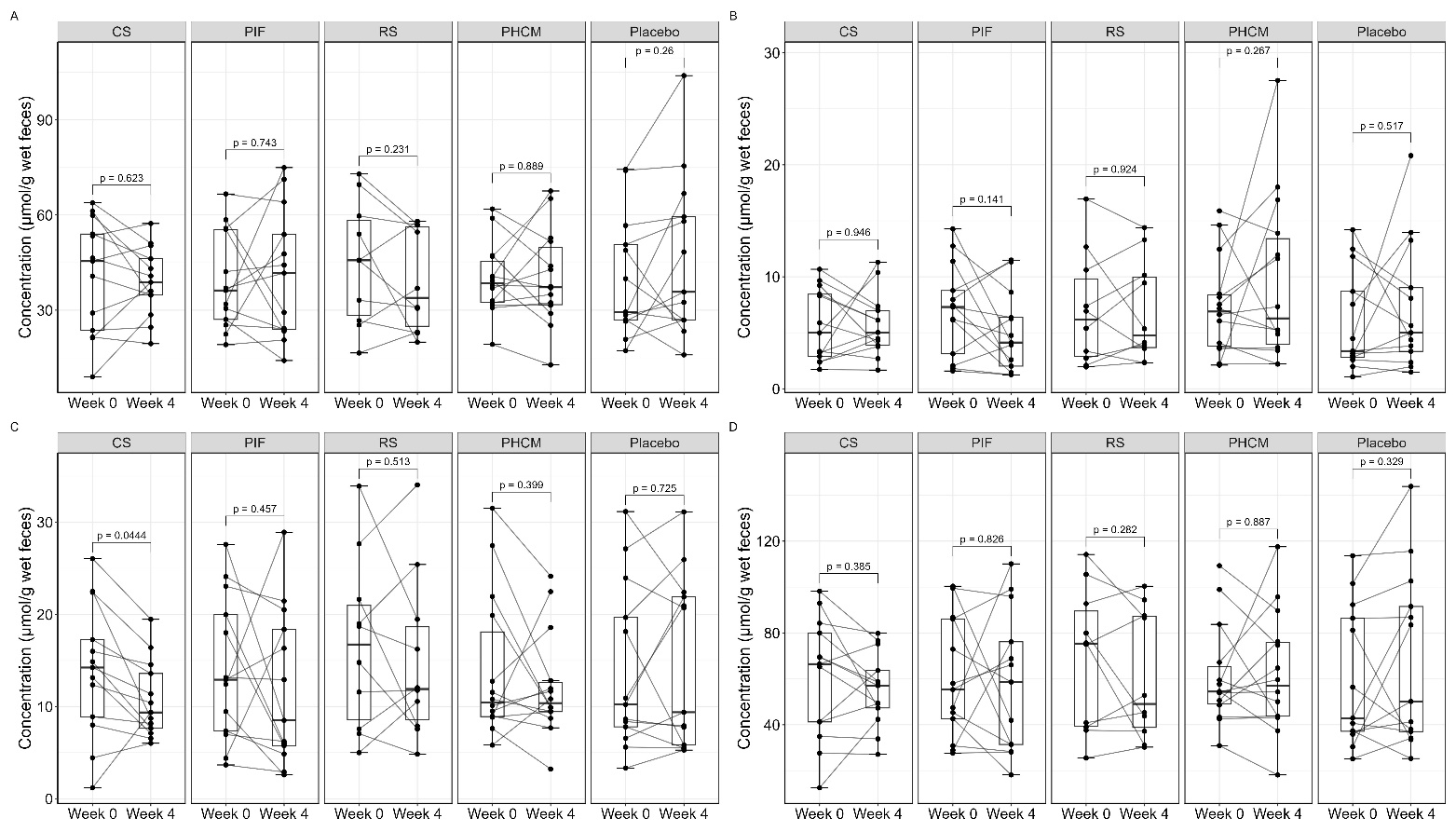


**Figure S5. Faecal short-chain fatty acid concentrations**Showing the concentration of Acetate (A), Propionate (B), Butyrate (C) an the total concentration of short-chain fatty acids (D), before (week 0) and after (week 4) supplementation with either CS, PIF, RS, PHCM or placebo.
